# Supplementary material for: Yiai Fuzheng decoction inhibits triple-negative breast cancer by remodeling the immune microenvironment
Source: Front Immunol. 2025 Sep 30;16:1615631. doi: 10.3389/fimmu.2025.1615631 (PMC12518410; doi:10.3389/fimmu.2025.1615631)
Supplement: Supplementary file 2 [file DataSheet1.zip › Supplementary File 2/Treatment/D_vs_H/07_Pathway_Analysis/Pathway_Analysis/Sig_Pathways_ID/Arginine and proline metabolism.pdf]

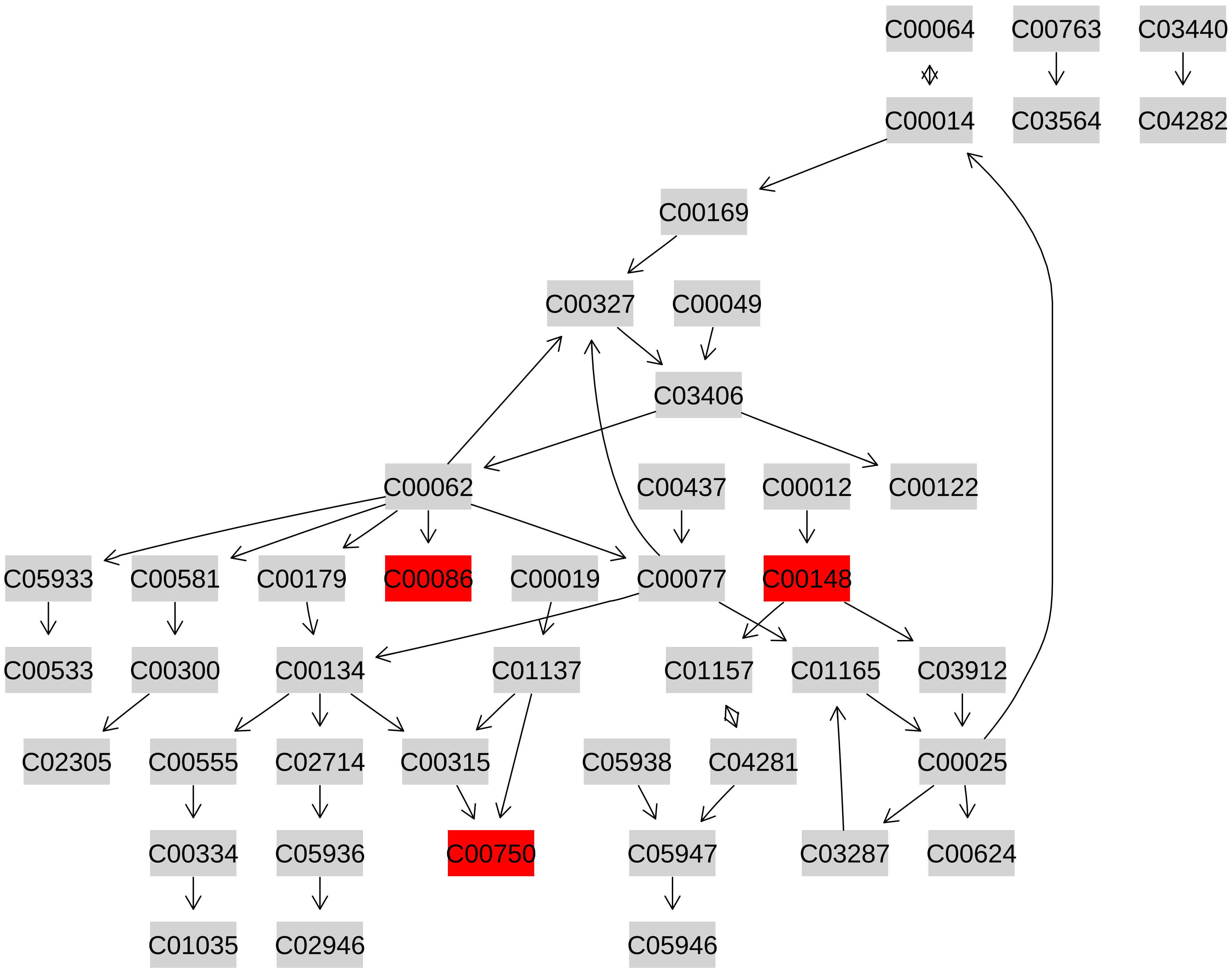
C00064
C00763
C03440
C00014
C03564
C04282
C00169
C00327
C00049
C03406
C00062
C00437
C00012
C00122
C05933
C00581
C00179
C00086
C00019
C00077
C00148
C00533
C00300
C00134
C01137
C01157
C01165
C03912
C02305
C00555
C02714
C00315
C05938
C04281
C00025
C00334
C05936
C00750
C05947
C03287
C00624
C01035
C02946
C05946
